# Effect of the Reduction Degree of Graphene Oxide on the Cyclization of Polyacrylonitrile in PAN/GO Composite Membranes Prepared by Phase Inversion

**DOI:** 10.3390/membranes16100345

**Published:** 2026-10-19

**Authors:** Ryszard Fryczkowski, Beata Fryczkowska

**Affiliations:** Institute of Engineering Sciences, University of Bielsko-Biała, Willowa 2, 43-309 Bielsko-Biała, Poland

**Keywords:** polyacrylonitrile, graphene oxide, reduced graphene oxide, PAN/GO membranes, phase inversion, thermal reduction, cyclization, DSC, FTIR, EDS, SEM

## Abstract

This study evaluates the effect of the reduction degree of graphene oxide on the cyclization of polyacrylonitrile (PAN) in composite PAN/GO membranes prepared by phase inversion. All composite membranes contained the same amount of graphene-based additive, namely 5 wt.% relative to dry PAN, which made it possible to separate the effect of additive loading from the effect of surface chemistry. The additives were obtained from a single batch of graphene oxide and subjected to controlled thermal reduction. Their oxygen contents, determined by EDS, were 7.2, 15.3, 24.6, 35.4 and 48.1 wt.% for PAN/GO-1, PAN/GO-2, PAN/GO-3, PAN/GO-4 and PAN/GO-5, respectively. DSC analysis showed that, in the PAN/GO-2-PAN/GO-5 series, the maximum temperature of the exothermic PAN cyclization effect decreased from 294 to 224 °C as the oxygen content increased, while the cyclization enthalpy decreased from 506.7 to 404.2 J/g. PAN/GO-1, containing the most strongly reduced additive with 7.2 wt.% oxygen, showed a different behavior, with the highest cyclization maximum, approximately 316 °C, and the highest enthalpy, 628.5 J/g. FTIR spectra confirmed the disappearance of the nitrile band at approximately 2240 cm^−1^ and the growth of C=N/C=C bands assigned to the developing PAN ladder structure. SEM observations showed that all selected membranes retained an asymmetric, macrovoid-containing architecture, while local differences in surface topography and cross-sectional heterogeneity were present. The results demonstrate a non-monotonic response across the complete series and indicate an association between reduction treatment, total oxygen content, and PAN cyclization; changes in dispersion, morphology, and polymer–filler interfacial organization may also contribute.

## 1. Introduction

Polymer membranes are among the most important functional materials used in separation, filtration, environmental, energy, and biotechnology processes. Polyacrylonitrile (PAN) occupies a special position among membrane-forming polymers because of its good chemical resistance, suitability for phase-inversion processing, and susceptibility to further physical and chemical modification. PAN is also an important precursor of carbon materials because it undergoes cyclization during thermal treatment, producing ladder structures that constitute an intermediate stage in stabilization and carbonization. The mechanism and kinetics of PAN stabilization and cyclization have been extensively studied by DSC and FTIR [1,2,3,4,5,6].

In recent years, considerable attention has been paid to composite membranes containing graphene-based additives, including graphene oxide (GO) and reduced graphene oxide (rGO). These materials may affect membrane morphology, hydrophilicity, mechanical and transport properties, antifouling performance, and thermal behavior. Previous studies by the authors on PAN/GO and rGO/PAN membranes demonstrated that GO or rGO significantly modifies the physicochemical, transport, and thermal properties of membranes prepared by phase inversion [7,8,9,10,11,12].

Graphene oxide contains numerous oxygen-containing groups, including hydroxyl, epoxy, carbonyl, and carboxyl groups. Reduction of GO partially removes oxygen and changes the character of the material surface. The authors’ experience and previous studies show that GO/rGO materials obtained under controlled procedures can be reliably characterized in terms of oxidation degree, elemental composition, structural properties, and interactions with a polymer matrix [7,8,13,14,15,16,17,18,19,20,21,22]. This knowledge was used as the basis for designing the comparative series of PAN/GO membranes investigated here.

Previous research on PAN/GO membranes has focused mainly on separation properties, porous structure, hydrophilicity, permeability, and fouling resistance. Beata Fryczkowska et al. showed that adding GO to PAN membranes prepared from PAN/DMF solutions by phase inversion affects membrane morphology, transport properties, fouling resistance, and PAN cyclization temperature [7]. A separate study demonstrated the hydrothermal reduction of GO to rGO directly within PAN membranes and confirmed the transformation by FTIR, Raman, WAXS, and TGA [8]. Gao et al. used XPS together with thermal and structural methods to show that GO–PAN interfacial reactions depend on the chemical state of the GO surface [23].

The present study uses a comparative experimental design. All composite membranes were prepared with the same graphene-based additive content, equal to 5 wt.% relative to dry PAN. The GO/rGO additives originated from one graphene oxide batch and were subjected to controlled thermal reduction to produce materials with different total oxygen contents. This design enables relative changes in PAN cyclization to be analyzed without simultaneously changing the filler content. In contrast to studies that vary the presence or loading of GO, the present work compares a reduction series obtained from the same initial GO batch at a fixed additive content and evaluates the resulting thermal response together with representative membrane morphology.

The aim of this study was to determine how the reduction degree of the graphene-based additive affects the maximum PAN cyclization temperature, cyclization enthalpy, and changes observed in FTIR spectra. The study covers PAN/GO membranes prepared by phase inversion and their thermal and spectroscopic responses. It builds on the authors’ previous experience with GO/rGO materials and focuses on the practical importance of additive reduction degree in the design of PAN/GO composite membranes. Representative surface and cross-sectional SEM images were additionally examined to determine whether morphological or dispersion-related effects could contribute to the thermal response.

## 2. Materials and Methods

### 2.1. Materials

The membranes were prepared using polyacrylonitrile in the form of a copolymer containing 93.9% acrylonitrile, 5.8% methyl acrylate, and 0.3% allyl methyl sulfonate. The polymer had a molecular weight Mw of approximately 85,000 g/mol. N,N-dimethylformamide (DMF) was used as the solvent, consistent with the previously applied methodology for preparing PAN and PAN/GO membranes by phase inversion from PAN/DMF solutions [7,14]. The graphene-based additives comprised GO and GO/rGO materials obtained from a single GO batch by controlled thermal reduction. The chemicals used in the study, including DMF and the reagents employed in the modified Hummers procedure, were of analytical grade and were used as received. The PAN copolymer is specified by its comonomer composition and molecular weight. Supplier information was not retained in the original experimental records and could not be verified retrospectively. Deionized water was used as the coagulation medium.

### 2.2. Preparation of GO/rGO Additives

The graphene-based additives were prepared from a single batch of graphene oxide obtained by a modified Hummers method, in accordance with the authors’ experience in GO synthesis and processing [13,24,25]. After purification and removal of acidic impurities, the starting material was thermally reduced at 300 °C for different periods to obtain materials with different degrees of reduction. Reduction was carried out under nitrogen flowing at 5 L/min to remove gaseous deoxygenation products and limit access of atmospheric oxygen. The material used in PAN/GO-5 was not reduced, whereas the additive used in PAN/GO-1 was reduced for the longest period and, consequently, had the lowest oxygen content in the series. The furnace was heated from room temperature to 300 °C at 10 °C/min; the holding times are reported in Table 1. After reduction, the samples were cooled under nitrogen.

The methodology was based on the authors’ previous studies, in which GO/rGO materials and polymer membranes containing graphene-based additives were characterized by FTIR, Raman, WAXS/XRD, TGA, SEM/EDS, DSC, and transport-property measurements [7,8,9,10,11,12,13,14,15,16,17,18,19,20,21,22,24,25,26,27,28]. These studies provide the methodological background for the present PAN/GO membrane series.

Using a single GO batch is methodologically important because it limits the variability associated with differences between independent syntheses. Consequently, comparison of PAN/GO-1-PAN/GO-5 primarily concerns the effects of reduction time, oxygen content, and reduction degree on PAN cyclization, while the origin and amount of the additive and the membrane-preparation procedure remain constant.

### 2.3. Preparation of PAN/GO Membranes

PAN/GO membranes were prepared by phase inversion using a uniform procedure previously applied to PAN/GO and PAN/PANI membranes [7,14]. The casting solution contained PAN in DMF at a concentration of 12 wt.% relative to the solvent. The appropriate GO/rGO additive was first dispersed in DMF and sonicated to obtain a homogeneous dispersion of graphene-based sheets. Dry PAN was then added, and the mixture was stirred until a homogeneous casting solution was obtained. The solution was cast onto a glass plate, after which the polymer layer was coagulated in water.

**Figure 1:**
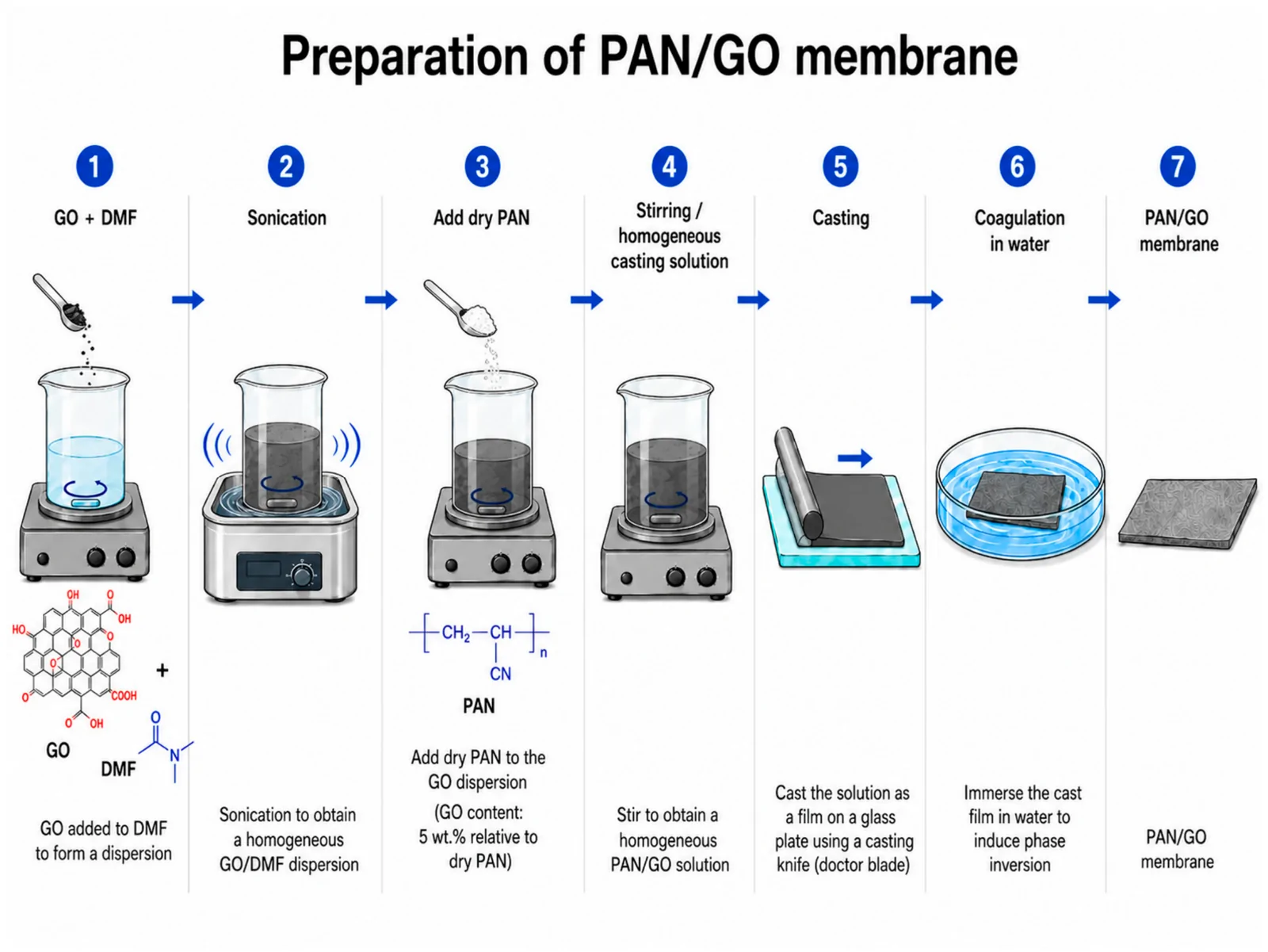
Scheme of PAN/GO membrane preparation by phase inversion. In all composite membranes, the graphene-based additive content was 5 wt.% relative to dry PAN.

All composite membranes contained 5 wt.% graphene-based additive relative to dry PAN. Maintaining the same additive content in all samples enabled the effect of reduction degree to be analyzed without interference from varying filler loading. The additive-free reference sample was designated PAN-0, while the composite membranes were designated PAN/GO-1, PAN/GO-2, PAN/GO-3, PAN/GO-4, and PAN/GO-5. The complete membrane-preparation procedure is illustrated in Figure 1.

### 2.4. DSC Characterization

PAN cyclization was analyzed by differential scanning calorimetry (DSC). The analysis focused on the maximum of the exothermic cyclization effect and on the cyclization enthalpy determined by integration of the exothermic peak area. This methodology is consistent with previous studies concerning the effect of GO additives on PAN cyclization in composite membranes [1,2,3,4,5,6,7,9,10,11,12].

Measurements were performed under nitrogen, enabling the effect of graphene-based additives on the exothermic course of PAN cyclization to be compared while limiting simultaneous oxidation by the gas phase. Enthalpy was expressed per gram of the membrane sample. Because all composite membranes contained the same amount of graphene-based additive, comparison of enthalpy values within the series is methodologically consistent.

DSC measurements were performed using a TA Instruments MDSC 2920 calorimeter (TA Instruments, New Castle, DE, USA). Samples were heated under nitrogen flowing at 40 mL/min from −40 to 320 °C at 10 °C/min. The DSC curves were analyzed using TA Instruments Universal Analysis V4.5A software. One DSC run was available for each membrane composition; consequently, the reported Tmax and enthalpy values are treated as descriptive results, and no statistical significance is claimed.

### 2.5. FTIR Characterization

Structural changes occurring during PAN cyclization were analyzed by FTIR spectroscopy. Particular attention was paid to the nitrile-group band at approximately 2240 cm^−1^ and to bands associated with C=N and C=C structures characteristic of the developing PAN ladder structure. The use of FTIR to evaluate GO/rGO and transformations in PAN membranes is consistent with the authors’ previous studies [1,4,6,7,8,16,17,18].

FTIR spectra were recorded using a Nicolet 6700 FT-IR spectrometer (Thermo Electron Corp., Madison, WI, USA) equipped with an MTEC model 300 photoacoustic accessory. Samples were placed in the photoacoustic analysis holder. Spectra were collected over 500–4000 cm^−1^ at a resolution of 4 cm^−1^ using 64 scans and a DTGS detector. Temperature-dependent measurements were conducted with an accessory enabling controlled sample heating from 20 to 300 °C with an accuracy of ±0.5 °C. Data were recorded and initially processed using OMNIC v. 8.0.

### 2.6. SEM Characterization

Representative membrane surfaces and cross-sections were examined by scanning electron microscopy. Cross-sections were prepared by fracturing the membrane specimens, and the samples were rendered electrically conductive before observation. Images were acquired at an accelerating voltage of 10 kV using a scanning electron microscope; the instrument model, manufacturer and coating details were not retained in the archived measurement records. SEM was used for qualitative comparison of membrane architecture, pore morphology, and the possible occurrence of local aggregates; no quantitative pore-size distribution or elemental identification of bright features was inferred from these images.

## 3. Results and Discussion

### 3.1. Experimental Design and Oxygen Content of the Graphene-Based Additives

The main experimental assumption was to determine the effect of the reduction degree of the graphene-based additive on PAN cyclization. Therefore, all composite membranes contained the same additive loading, 5 wt.% relative to dry PAN, and GO/rGO materials obtained by controlled thermal reduction of one GO batch. This design limited variability associated with filler amount and origin, allowing differences observed by DSC and FTIR to be analyzed in relation to the reduction degree of the graphene-based materials.

The use of a single initial GO batch was particularly important because individual syntheses may produce materials differing in oxidation degree, oxygen content, defect density, dispersibility, and ability to interact with the polymer matrix. Previous studies of GO/rGO, PAN/GO membranes, and polymer composites confirm that the properties of these materials depend on the synthesis route, reduction conditions, and method used to introduce the additive into the polymer [7,8,13,14,15,16,17,18,19,20,21,22,24,25].

The oxygen content of the graphene-based materials ranged from 7.2 to 48.1 wt.%. The lowest value was found for the additive used in PAN/GO-1, whereas the highest was found for the material used in PAN/GO-5. Increasing oxygen content corresponded to a decrease in the GO/rGO reduction degree.

**Figure 2:**
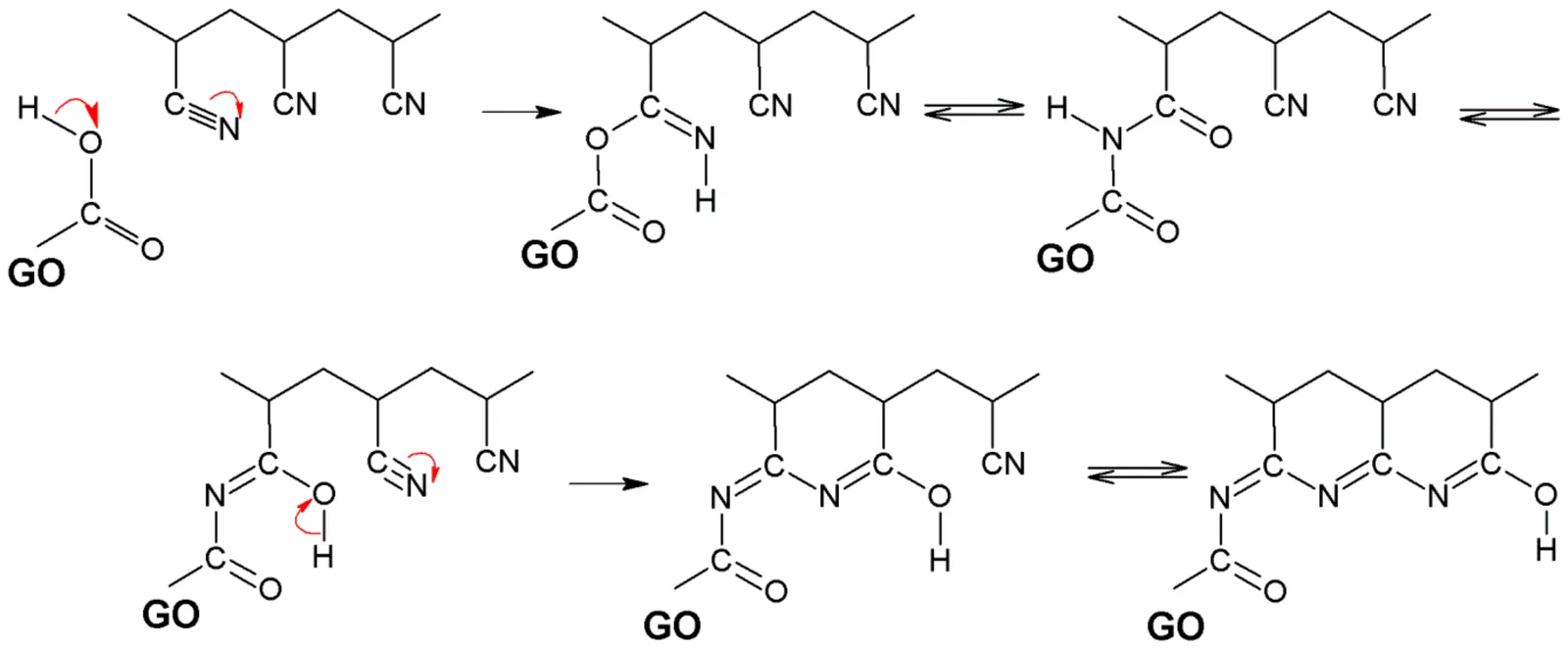
Proposed scheme for the participation of oxygen-containing GO functional groups in the initiation of polyacrylonitrile cyclization.

Total oxygen content was adopted as a parameter describing the oxidation or reduction degree of the graphene-based additive. This parameter affects interactions with PAN, dispersion in DMF, and behavior during heating. For materials obtained from one GO batch, it indicates the direction of changes occurring during reduction and enables additive properties to be correlated with the thermal and spectroscopic responses of the membranes. It should be emphasized, however, that EDS determines total oxygen content but does not identify individual functional groups [16,17,18,20,21,22]. The proposed role of oxygen-containing GO groups in PAN cyclization is illustrated schematically in Figure 2.

### 3.2. SEM Morphology of the Membranes

**Figure 3:**
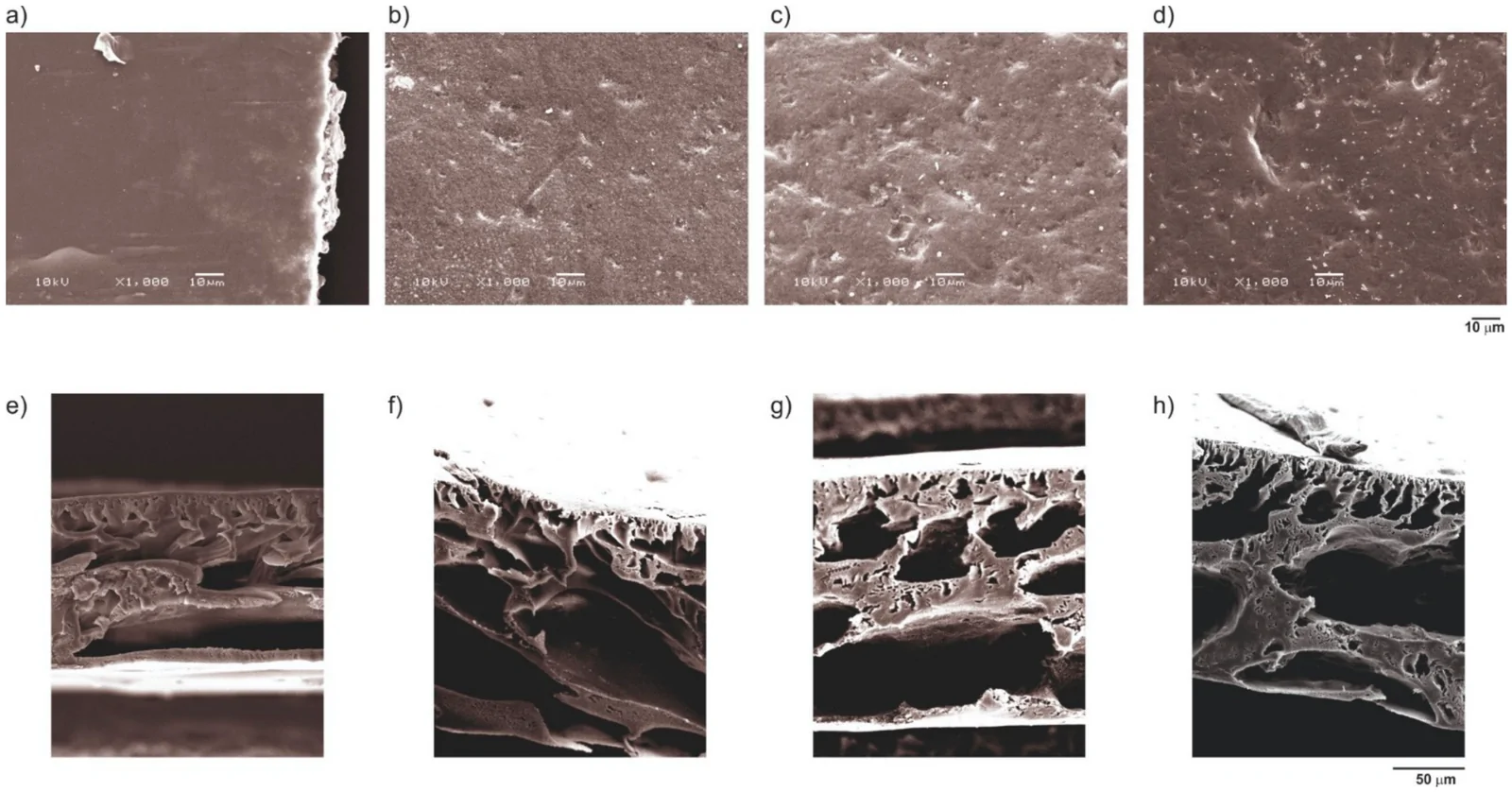
Representative SEM images of the membrane surfaces (**a**–**d**; ×1000) and corresponding cross-sections (**e**–**h**; ×500): PAN-0 (**a**,**e**), PAN/GO-1 (**b**,**f**), PAN/GO-3 (**c**,**g**), and PAN/GO-5 (**d**,**h**). The cross-sections are oriented horizontally, with the upper compact surface layer positioned at the top.

Representative SEM images of PAN-0, PAN/GO-1, PAN/GO-3 and PAN/GO-5 are presented in Figure 3. The surface images were recorded at ×1000, while all cross-sectional images were recorded at ×500 and oriented horizontally with the upper, comparatively compact membrane surface positioned at the top. The cross-sections reveal an asymmetric porous architecture comprising a comparatively compact surface region and a macrovoid-rich substructure. This general arrangement is retained in both the PAN-0 membrane and the membranes containing graphene-based additives.

At ×1000, the PAN-0 surface appears relatively smooth and compact, whereas the composite-membrane surfaces exhibit more heterogeneous topographies with locally distributed brighter features. Because image contrast alone does not establish the chemical identity of these features, no compositional interpretation was assigned to them. The surface micrographs therefore indicate local topographical heterogeneity but do not provide evidence of the nanoscale distribution of the graphene-based additives.

The cross-sectional images show irregular and elongated macrovoids separated by polymer walls containing finer porous regions. Differences are visible in macrovoid shape, size, and spatial distribution, as well as in the local thickness and continuity of the polymer walls. However, the images do not demonstrate a systematic change in cross-sectional morphology with increasing oxygen content of the graphene-based additive. Accordingly, the SEM observations are treated as qualitative and show that the overall asymmetric membrane architecture was preserved throughout the selected series.

### 3.3. DSC Analysis of PAN Cyclization in PAN/GO Membranes

**Figure 4:**
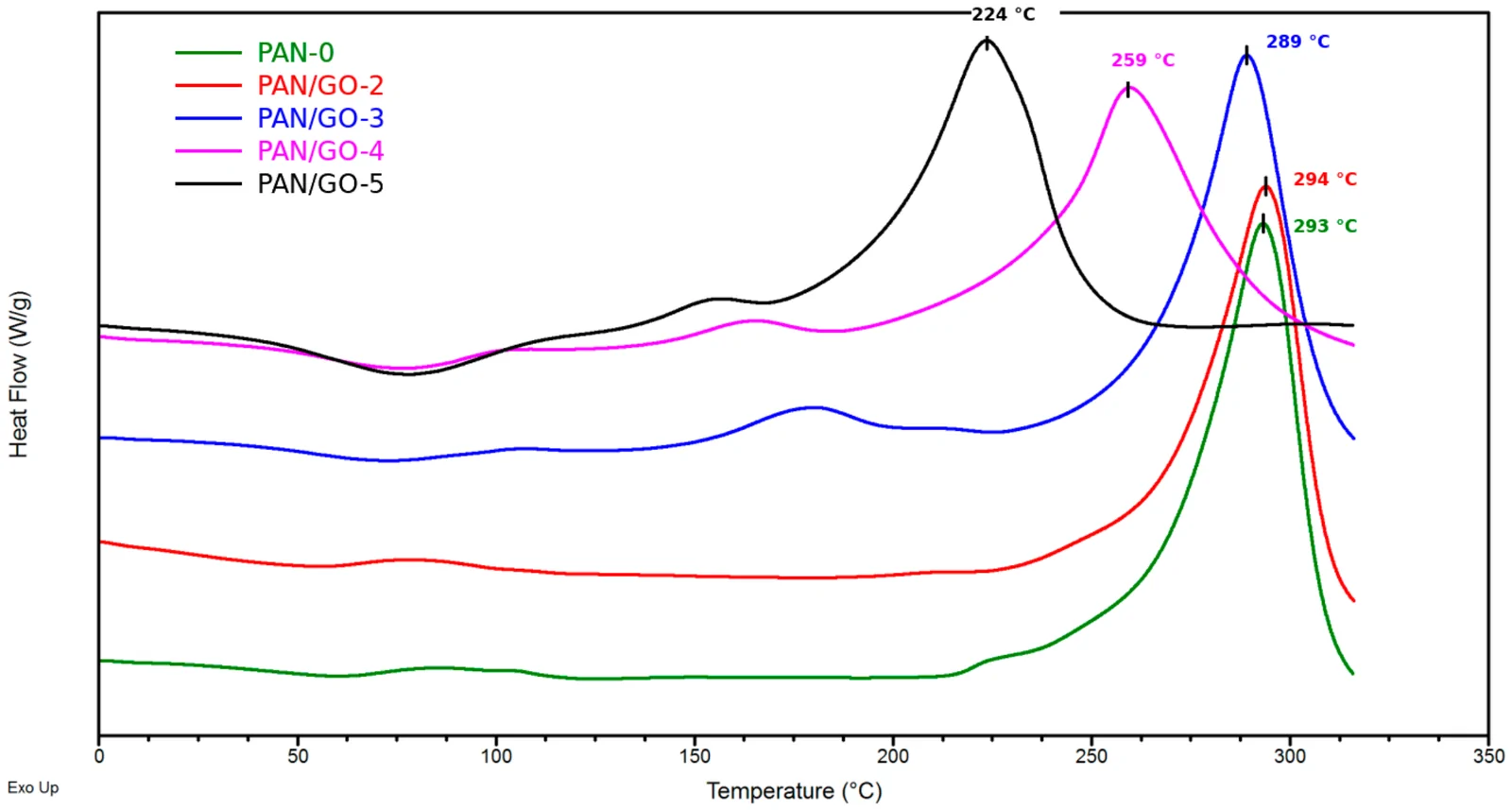
DSC curves for PAN-0 and PAN/GO-2-PAN/GO-5. With increasing oxygen content in the graphene-based additive, the maximum of the exothermic cyclization effect shifts toward lower temperatures.

DSC analysis revealed clear differences in PAN cyclization depending on the graphene-based additive used. In the PAN/GO-2-PAN/GO-5 series, the maximum temperature of the exothermic cyclization effect decreased systematically as the oxygen content of the additive increased. The maximum temperature decreased from 294 °C for PAN/GO-2 to 224 °C for PAN/GO-5, while the cyclization enthalpy decreased from 506.7 to 404.2 J/g. The corresponding DSC curves are presented in Figure 4.

**Figure 5:**
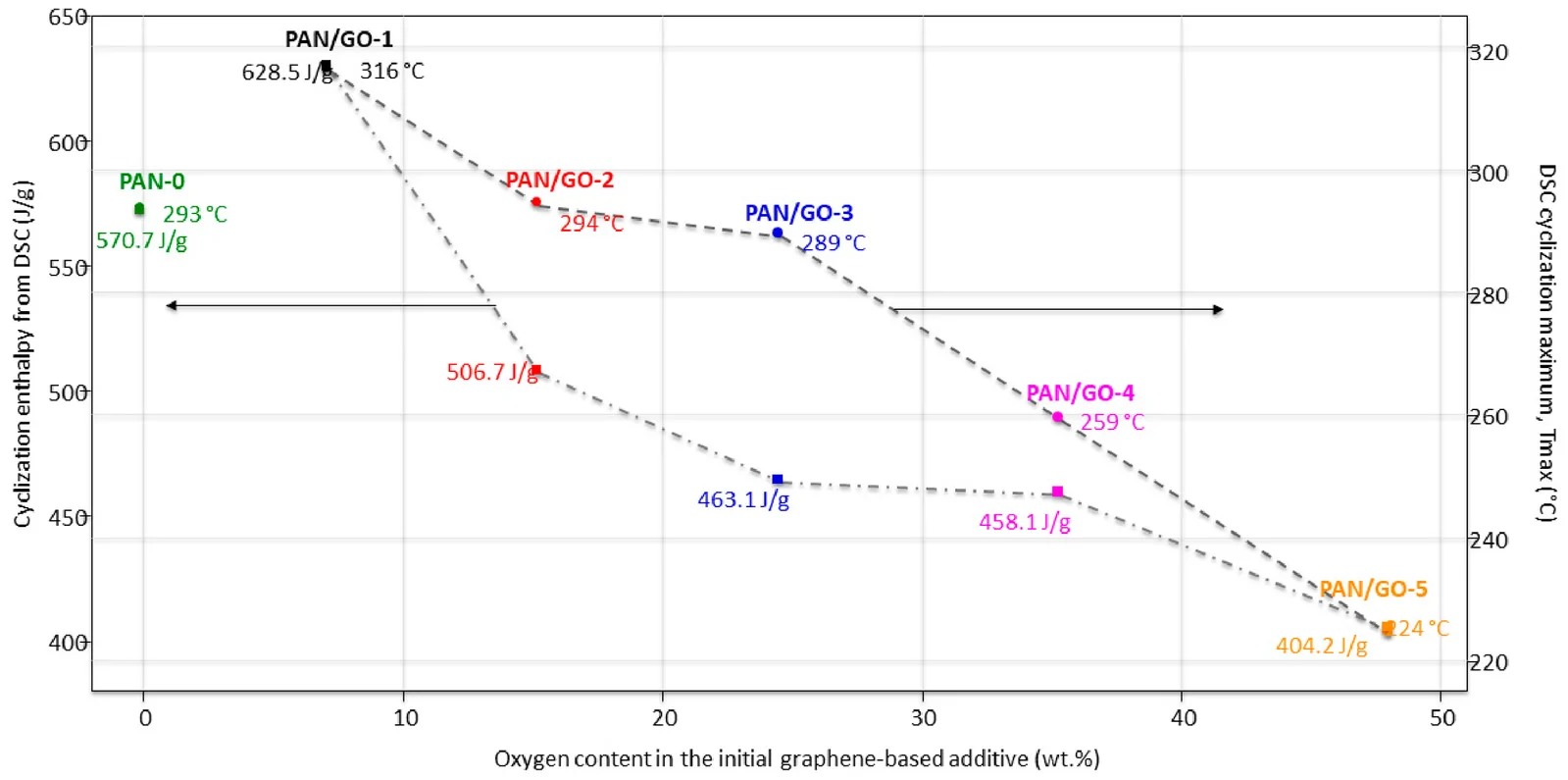
Dependence of the maximum temperature of the PAN cyclization DSC effect (left y-axis) and cyclization enthalpy (right y-axis) on the oxygen content of the initial graphene-based additive. The PAN-0 reference point is shown separately and is not connected to the PAN/GO series.

Within PAN/GO-2–PAN/GO-5, increasing total oxygen content was associated with a shift in the cyclization maximum toward lower temperatures. However, the response was not linear: Tmax changed by only 5 °C between 15.3 and 24.6 wt.% O, but by 30 and 35 °C over the next two intervals. PAN/GO-2 differed from PAN-0 by only 1 °C. These results are therefore more consistent with a nonlinear or threshold-like response than with a simple proportional oxygen-content effect. Because independent replicate membranes and replicate DSC runs were not available, differences such as PAN-0 versus PAN/GO-2 and PAN/GO-2 versus PAN/GO-3 cannot be assigned statistical significance. Total oxygen measured by EDS does not identify hydroxyl, epoxy, carbonyl, or carboxyl populations; XPS would be required to test a functional-group-specific mechanism [16,17,18,20,21,22,23]. Similar GO-related changes in PAN stabilization have been reported for PAN/GO composites, nanofibers, and fibers [9,10,11,12], although differences in processing and morphology limit direct comparison. Figure 5 summarizes the dependence of both DSC parameters on the oxygen content of the graphene-based additive.

The maximum DSC temperatures and cyclization enthalpies are summarized in Table 2. The values describe a decreasing sequence across PAN/GO-2–PAN/GO-5 and the distinct behavior of PAN/GO-1, but they should not be interpreted as a statistically validated monotonic trend. Figure 5 summarizes the dependence of Tmax and cyclization enthalpy on the oxygen content of the graphene-based additive. A contextual comparison with the selected PAN/GO literature is provided in Table 3.

This comparison is not intended to claim that the present membranes outperform all previously reported PAN/GO systems. Instead, it shows that the principal contribution of this work is the controlled variation of oxygen content in additives obtained from one GO batch while maintaining a constant filler loading.

**Figure 6:**
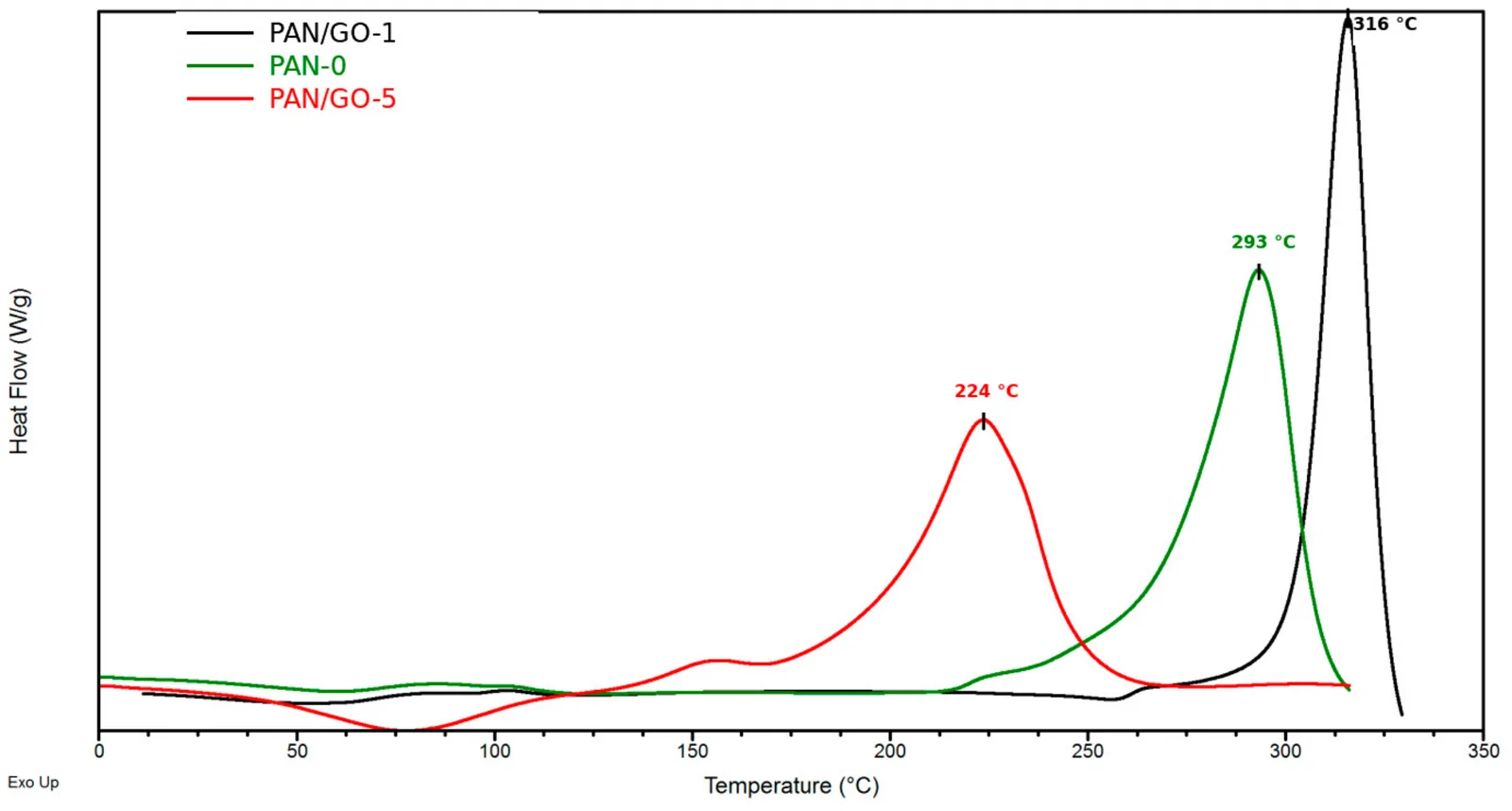
Comparison of DSC curves for PAN/GO-1, PAN-0 and PAN/GO-5.

PAN/GO-1, containing the most strongly reduced additive with 7.2 wt.% oxygen, behaved differently. Its cyclization maximum was approximately 316 °C and its cyclization enthalpy was 628.5 J/g. This is not a minor deviation but a reversal relative to the PAN/GO-2–PAN/GO-5 sequence. Extensive thermal reduction can alter not only oxygen content but also defect density, sheet stacking, accessible surface area, dispersibility in DMF, and polymer–filler interfacial organization. Any of these factors, individually or jointly, could reduce the availability of reactive interfacial sites or change local chain mobility and thereby contribute to the higher Tmax and enthalpy of PAN/GO-1. The present data do not allow this response to be assigned exclusively to residual oxygen functionality. A direct comparison of PAN/GO-1, PAN-0 and PAN/GO-5 DSC curves is shown in Figure 6.

The behavior of PAN/GO-1 indicates that the relationship between oxygen content and PAN cyclization is not linear over the entire investigated range. The SEM images confirm retention of an asymmetric porous architecture but cannot establish the nanoscale distribution of the strongly reduced additive. Therefore, dispersion and interfacial contact should be considered together with reduction degree when interpreting the anomalous PAN/GO-1 response.

### 3.4. FTIR Analysis of Structural Changes in PAN

**Figure 7:**
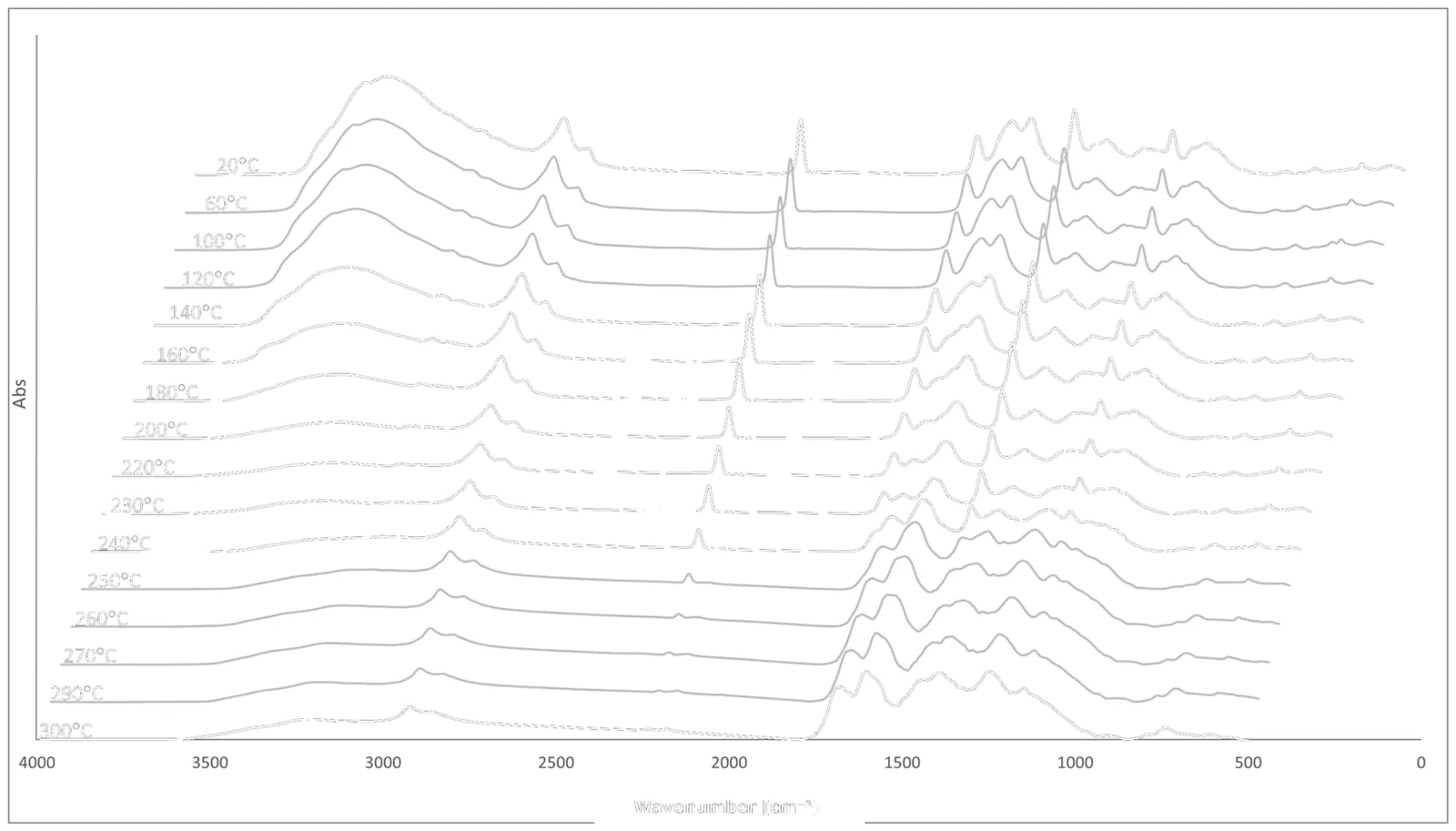
FTIR spectra of the PAN/GO-5 membrane recorded as a function of temperature.

FTIR spectra confirmed structural changes characteristic of PAN cyclization. In particular, the intensity of the nitrile-group band at approximately 2240 cm^−1^ decreased, while the intensity of bands associated with C=N and C=C structures increased. These changes are consistent with the formation of the conjugated PAN ladder structure during thermal treatment. The temperature-dependent spectra of PAN/GO-5 are presented in Figure 7.

The FTIR results complement the DSC analysis by confirming the chemical transformations characteristic of PAN cyclization. However, the temperature-dependent FTIR series was obtained for PAN/GO-5 and was not recorded comparably for all reduction states. It therefore demonstrates that cyclization occurs in PAN/GO-5 but does not establish systematically different chemical pathways across PAN/GO-1–PAN/GO-5 and does not directly explain the anomalous PAN/GO-1 response.

### 3.5. Comparison of FTIR Spectra of the Components and the Heated Membrane

**Figure 8:**
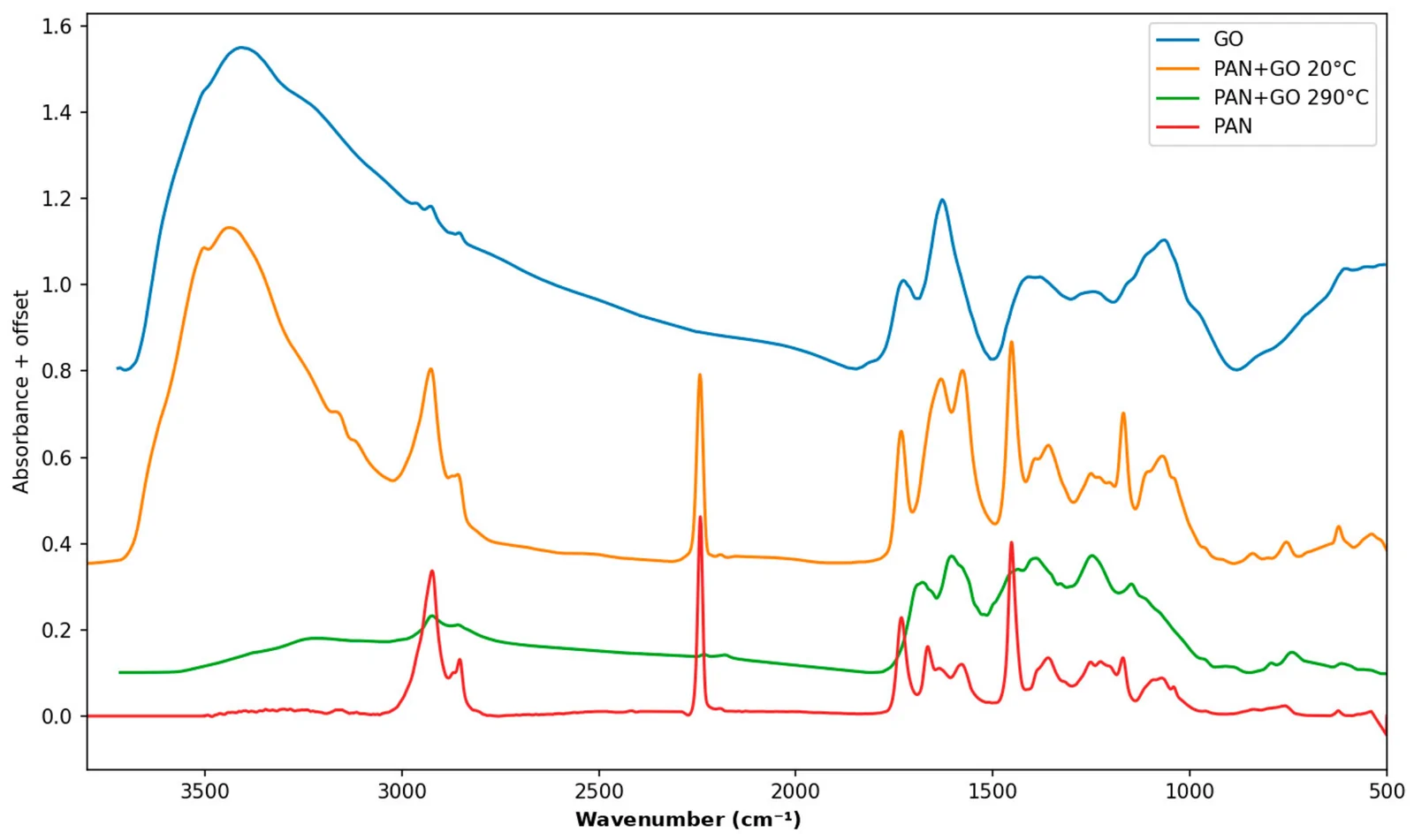
FTIR spectra of the initial GO, neat PAN, and the PAN/GO-5 membrane at 20 °C and after heating to 290 °C.

The spectra of the starting GO, neat PAN, and PAN/GO-5 before and after heating are compared in Figure 8.

DSC and FTIR provide complementary thermal and structural information. Their qualitative consistency supports assignment of the observed exotherm to PAN cyclization, but it should not be interpreted as proof that total oxygen content or a specific oxygen functional group is the sole controlling variable.

### 3.6. Significance of the Results for PAN/GO Membranes

The results are relevant to the design of PAN/GO membranes, particularly when the membranes are intended for further thermal treatment or use as precursors of stabilized or partially carbonized structures. Lowering the PAN cyclization maximum may reduce the required treatment temperature and energy consumption and improve control over structural transformations.

The results show that membrane thermal behavior depends not only on the presence of the graphene-based additive but also on its reduction treatment. At constant loading, total oxygen content was associated with substantial differences in Tmax and enthalpy, but the full response was non-monotonic and may include contributions from pore morphology, dispersion, and interfacial contact. Because DSC was performed under flowing nitrogen, membrane porosity is not interpreted here in terms of gas-phase oxygen accessibility. Morphology may nevertheless influence heat transfer, local polymer packing, and the extent of polymer–filler contact during heating.

From the perspective of a membrane-focused journal, the study concerns not merely graphene-based nanomaterials but PAN/GO composite membranes prepared by phase inversion. The results relate directly to the behavior of these membranes during heating and may support the development of membranes with controlled thermal and structural properties.

## 4. Conclusions

The reduction degree and total oxygen content of the graphene-based additive were associated with changes in PAN cyclization in PAN/GO composite membranes prepared by phase inversion. All membranes contained the same additive amount, 5 wt.% relative to dry PAN, which allowed differences between additives to be evaluated at constant filler loading.

In PAN/GO-2-PAN/GO-5, increasing the oxygen content of the graphene-based additive from 15.3 to 48.1 wt.% decreased the PAN cyclization maximum from 294 to 224 °C and reduced the cyclization enthalpy from 506.7 to 404.2 J/g. PAN/GO-1, containing the most strongly reduced additive with 7.2 wt.% oxygen, behaved differently and showed the highest cyclization maximum, approximately 316 °C, and the highest enthalpy, 628.5 J/g.

FTIR analysis of PAN/GO-5 confirmed the structural transformations associated with PAN cyclization, particularly the decrease in the nitrile-group band and the increase in bands assigned to C=N/C=C structures. These spectra support the assignment of the thermal transformation but do not demonstrate different chemical pathways for all reduction states. SEM showed that the selected membranes retained an asymmetric macrovoid-containing structure, with qualitative differences in surface topography and cross-sectional heterogeneity.

The findings show that the PAN/GO membrane design should account not only for the amount of graphene-based additive but also for its reduction degree. This observation is relevant to the development of PAN/GO composite membranes with controlled thermal behavior and to further studies of the stabilization and carbonization of these systems. The anomalous PAN/GO-1 response and the absence of replicate DSC/EDS measurements mean that the observed relationship should be treated as descriptive rather than as a statistically validated universal dependence. Further work should combine replicate membrane preparation and DSC with complementary surface-chemical and structural characterization to separate the effects of oxygen functionality, dispersion, and interfacial organization.

## Figures and Tables

**Table 1:** Composition of PAN/GO membranes and oxygen content of the initial graphene-based additives determined by EDS. The reported EDS oxygen contents are single determinations; replicate variability was not available.

Sample	PAN (wt.%)	GO/rGO Additive (wt.%)	Reduction Time at 300 °C (min)	Oxygen Content by EDS (wt.%)
PAN-0	100	0	-	-.
PAN/GO-1	95	5	60	7.2
PAN/GO-2	95	5	30	15.3
PAN/GO-3	95	5	15	24.6
PAN/GO-4	95	5	5	35.4
PAN/GO-5	95	5	0	48.1

**Table 2:** Maximum temperature of the DSC effect and cyclization enthalpy of PAN and PAN/GO membranes. Values represent single DSC determinations for each composition.

Sample	Oxygen Content in the GO Additive by EDS (wt.%)	DSC Maximum, Tmax (°C)	Cyclization Enthalpy from DSC (J/g)
PAN-0	-	293.0	570.7
PAN/GO-1	7.2	316.0	628.5
PAN/GO-2	15.3	294.0	506.7
PAN/GO-3	24.6	289.0	463.1
PAN/GO-4	35.4	259.0	458.1
PAN/GO-5	48.1	224.0	404.2

**Table 3:** Contextual comparison of the present study with selected literature reports on PAN/GO systems.

System/Reference	Main Variable	Main Characterization Focus	Relation to the Present Work
GO/PAN composites [9]	Presence of thermally reduced GO	DSC-based cyclization behavior	Shows that GO can affect PAN cyclization; does not isolate oxygen content at constant loading.
PAN/GO nanofibers [10]	GO in electrospun PAN fibers	Spectroscopic and electrochemical stabilization study	Addresses PAN/GO stabilization, but fiber morphology and processing strongly affect the system.
GO-coated PAN fibers [11]	GO coating on PAN fibers	Pre-oxidation and structural changes	Demonstrates GO-assisted pre-oxidation in fibers rather than phase-inversion membranes.
PAN/GO nanofibrous membrane [12]	GO-induced thermal oxidation	Heat resistance and flame retardancy of a filtration membrane	Focuses on high-temperature performance without independently analyzing oxygen content and additive loading.
This work	Reduction degree and oxygen content of one GO batch at constant 5 wt.% loading	DSC Tmax, cyclization enthalpy and FTIR changes	Examines the association between reduction treatment, total oxygen content and PAN cyclization at constant loading, while recognizing morphology and interfacial organization as possible co-variables.
PAN membranes from NMMO [26]	Active solvent system and PAN copolymer processing	Membrane fabrication from PAN/NMMO solutions	Presents an alternative PAN membrane route that differs from the PAN/DMF/GO system used here.
PAN/NMMO dissolution and thermal treatment [27,28]	PAN dissolution and spinning environment	Dissolution kinetics, spinning and thermal treatment of PAN precursors	Provides complementary processing context that is mechanistically different from GO-assisted cyclization.
Reduced GO membranes and GO reduction studies [19,20,21,22]	Reduction degree, C/O ratio and structural evolution of GO	Permeance, reduction mechanism, XRD, TEM and electron spectroscopy	Supports the conclusion that GO reduction changes oxygen quantity, surface chemistry, and material structure.
Graphene-based polymer composite [29]	Graphene-based additive in a polymer matrix	Composite structure and functional properties	Shows the need to consider morphology when interpreting graphene/polymer composite properties.
GO/PAN composite films [23]	GO–PAN interfacial chemistry	XPS, DSC, FTIR, Raman and XRD	Shows that PAN–GO interactions depend on interfacial chemistry and that total oxygen content alone cannot identify the reacting oxygen functionalities.

## Data Availability

The original contributions presented in this study are included in the article. Further inquiries can be directed to the corresponding author.
